# CDK4/6 Inhibitors Plus Endocrine Therapy in Early-Stage HR+/HER2− Breast Cancer: Updated Meta-Analysis of Phase III Trials †

**DOI:** 10.3390/cancers17213538

**Published:** 2025-11-01

**Authors:** Stamatia Alexiou, Georgios Mavrovounis, Georgios Christodoulopoulos, Stamatia Perifanou, Emmanouil Saloustros

**Affiliations:** 1 Department of Internal Medicine, General Hospital of Larissa, 41221 Larissa, Greece; stamalexiou@uth.gr; 2 Department of Emergency Medicine, Faculty of Medicine, School of Health Sciences, University of Thessaly, 41500 Larissa, Greece; gmavrovounis@gmail.com; 3 Division of Oncology, Faculty of Medicine, School of Health Sciences, University of Thessaly, 41500 Larissa, Greece

**Keywords:** early-stage HR+/HER2− breast cancer, CDK4/6 inhibitors, endocrine therapy, adjuvant therapy, meta-analysis, invasive disease-free survival, abemaciclib, ribociclib, palbociclib

## Abstract

**Simple Summary:**

HR+/HER2− breast cancer is a common type of early-stage breast cancer. Although endocrine therapy is the standard treatment, some patients still experience recurrence. This study updates previous analyses on adding CDK4/6 inhibitors (CDK4/6i) to endocrine therapy (ΕΤ) using the latest clinical trial data. The analysis includes data from randomized phase III studies with different patient risk profiles and treatment durations, providing a comprehensive overview of recent data. The findings contribute to the ongoing discussion about optimizing adjuvant therapy and may help refine treatment strategies and inform future research in early breast cancer.

**Abstract:**

Background/Objectives: This meta-analysis aimed to evaluate the efficacy of combining CDK4/6i with ET, compared with ET alone, in improving invasive disease-free survival (iDFS), distant recurrence-free survival (DRFS), and overall survival (OS) in early-stage hormone receptor-positive (HR+), human epidermal growth factor 2-negative (HER2−) breast cancer. Given the inconclusive findings of previous meta-analyses, an updated synthesis of the latest phase III trial data was performed. Methods: A systematic review and meta-analysis were conducted following PRISMA guidelines. Randomized Controlled Trials (RCTs) comparing CDK4/6i plus ET versus ET alone were identified through PubMed, Scopus, and ClinicalTrials.gov. Hazard ratios and adverse events were analyzed using appropriate statistical models. Results: Four RCTs (monarchE, NATALEE, PENELOPE-B, PALLAS) including 17,749 patients were analyzed. CDK4/6 inhibitors improved iDFS (HR 0.80; 95% CI: 0.67–0.96; *p* = 0.01), while a strong trend toward improved DRFS was observed (HR 0.79; 95% CI: 0.61–1.02; *p* = 0.07), suggesting a potential clinically relevant benefit that requires longer follow-up to confirm. The effect on OS (HR 0.95; 95% CI: 0.79–1.16; *p* = 0.63) remains inconclusive. Adverse events, including neutropenia and diarrhea, were more frequent with CDK4/6i. Conclusions: The addition of CDK4/6i to ET improves iDFS and shows a favorable trend in DRFS in early-stage HR+/HER2− breast cancer, highlighting the need for longer follow-up to clarify their long-term benefit.

## 1. Introduction

Breast cancer represents a major public health issue both globally and in Europe. In 2020, it was the most frequently diagnosed cancer in the European Union (EU-27), accounting for 13.3% of all newly diagnosed cancer cases and 28.7% of all cancers in women [1]. In 2020, breast cancer was the most frequently diagnosed cancer worldwide [2]. Among these, HR+/HER2− breast cancer remains the main type, comprising approximately 70% of all breast cancer diagnoses [3]. These data underscore the importance of advancing therapeutic strategies focused on this molecular subtype.

Among other therapeutic approaches, CDK4/6i has become one of the most important therapeutic advancements in breast cancer. Cyclin-dependent kinases (CDKs) are serine/threonine protein kinases that play a critical role in cell-cycle regulation, with their discovery marking a turning point in our understanding of breast cancer biology [4]. In estrogen receptor positive (ER+) breast cancer, activation of the ER signaling pathway leads to upregulation of the ER-cyclin D-CDK4/6 pathway. Specifically, the cyclin-CDK4/6 complex phosphorylates the retinoblastoma protein (Rb), releasing the transcription factor E2F and driving cell-cycle progression from the G1 to the S phase [5]. Consequently, it has been hypothesized that dual targeting of the ER and cyclin D–CDK4/6 INK4–Rb pathways can achieve more robust tumor inhibition and mitigate the development of endocrine resistance.

This therapeutic approach has laid the foundation for the clinical adoption of the combination of CDK4/6i with endocrine therapy as the standard of care for HR+/HER2− advanced or metastatic breast cancer [6]. Agents such as abemaciclib, ribociclib and palbociclib are now widely utilized, as they have demonstrated robust evidence of improving PFS and OS in advanced or metastatic breast cancer [7]. Expanding on these successes, attention has shifted to their role in earlier stages of HR+/HER2− breast cancer, where the opportunity to intervene before metastasis emerges as a promising strategy. As adjuvant therapy, these agents aim to enhance long-term outcomes, including DFS and OS, by eliminating any cancer cells that may remain after initial treatment and lowering the risk of cancer recurrence. This approach relies on the hypothesis that early intervention with CDK4/6 inhibitors can interrupt the cell cycle in dormant or proliferative cancer cells, which helps prevent progression to overt metastatic disease. Furthermore, these agents may enhance the efficacy of endocrine therapy by targeting mechanisms of resistance and providing a more effective overall treatment strategy [8,9]. Although CDK4/6 inhibitors have become a mainstay in the metastatic setting, their role in early breast cancer continues to be explored, as variations in study design, patient characteristics, and pharmacologic properties may contribute to differing results.

Multiple phase III randomized trials have examined the role of CDK4/6i in early-stage HR+/HER2− breast cancer with variable results. The monarchE trial showed prolonged efficacy in iDFS and DRFS with abemaciclib, whereas NATALEE demonstrated a consistent but more modest benefit with ribociclib. In contrast, both PALLAS and PENELOPE-B, which assessed palbociclib, did not demonstrate a significant iDFS advantage over endocrine therapy. Notably, these trials of adjuvant CDK4/6i differ in inclusion criteria, patient risk profiles, and treatment duration, factors that could influence the extent of benefit observed. These aspects are further examined in Section 3 [10,11,12,13].

A previous meta-analysis showed promising results, demonstrating that the combination of CDK4/6i with endocrine therapy improved iDFS in patients with early-stage HR+/HER2− breast cancer compared with those who were treated with hormone therapy alone [14]. Consequently, the objective of this meta-analysis is to re-evaluate these findings, incorporating updated data from clinical trials that became available after the publication of the aforementioned meta-analysis and to investigate whether particular agents or subgroups might explain variations in efficacy, acknowledging the evolving clinical landscape and recent regulatory approvals.

## 2. Materials and Methods

The protocol for this meta-analysis was developed in accordance with the PRISMA guidelines [15] (Table S1) and is available online on the International Prospective Register of Systematic Reviews (PROSPERO ID: CRD 42024612797). The full protocol is available online at: https://www.crd.york.ac.uk/prospero/display_record.php?RecordID=612797; accessed on 26 October 2025.

### 2.1. Literature Search

The PubMed, Scopus and clinicaltrials.gov databases were searched by two independent authors (A.S., M.G.). The following keywords were used combined with the Boolean operators “AND” and “OR”, as appropriate: breast neoplasm, breast tumor, breast cancer, mammary cancer, malignant neoplasm of breast, malignant tumor of breast, human mammary carcinomas, human mammary neoplasms, breast carcinoma, early stage, local advanced, non-metastatic, stage II, stage III, hormone receptor-positive, Human epidermal growth factor receptor 2-negative, Progesterone Receptor, Estrogen Receptors, Estrogen Nuclear Receptor, Cyclin-Dependent Kinase Inhibitor Proteins, CDKI Proteins, Cyclin-Kinase Inhibitor Proteins, CIP KIP, Cyclin Dependent Kinase Inhibitors, CIP KIP CKI Proteins, INK4 Cyclin Dependent Kinase Inhibitors, CKI Proteins, INK4, Inhibitors of Cyclin-Dependent Kinase 4 Protein, Protein Kinase Inhibitors, CDK-4 CDK-6, Abemaciclib, Palbociclib, Ribociclib, Cyclin Dependent Kinase 4, Cdk4 Cyclin-Dependent Kinase, Cdk4 Protein, Cdk4 Protein Kinase, p34PSK-J3 Kinase Cell Division Protein Kinase 4, PSK-J3 Kinase, Cyclin D-Dependent Kinase CDK4, Cyclin Dependent Kinase 6, Cell Division Protein Kinase 6, Cdk6 Protein Kinase, PLSTIRE Protein, Aromatase Inhibitors, Tamoxifen, SERM—selective estrogen receptor modulator, Hormone Therapy. The last literature search was performed on 15 November 2024.

### 2.2. Inclusion Criteria

The following criteria were used to identify the included studies: (1) RCTs, (2) comparing the administration of CDK4/6i plus ΕΤ, (3) versus ΕΤ alone, (4) in patients with early-stage HR+/HER2− breast cancer (Stage I–III). Regarding publication type, we elected to include (5) full text publications or conference abstracts. This decision was made to ensure that the latest available data were included in the review. We included only studies/abstracts written in the English language. No specific restrictions were used for follow-up duration and publication date.

### 2.3. Outcomes of Interest

Τhe primary outcomes of interest were iDFS, DRFS and OS. Side effects were identified as secondary outcomes of interest in the protocol. A qualitative synthesis of adverse events was performed based on the available data from each trial. However, a quantitative meta-analysis was not feasible due to heterogeneity in the reporting of adverse events, grading systems, and completeness of data across studies.

### 2.4. Data Extraction

As per the protocol, the following data were extracted from all studies: first author name, year of publication, age of patients, male to female ratio, the number of participants in each treatment arm, duration and details of therapy regimen, type of treatment in each arm, follow-up time, side effects, pre-menopausal to post-menopausal ratio, grade of malignancy, lymph node status, Ki67, definitions used for iDFS/DRFS/OS.

### 2.5. Quality of Studies and Publication Bias

The risk of bias of the studies was assessed using the Risk of Bias 2.0 tool for RCTs [16]. Publication bias was evaluated by visual inspection of the funnel plots for significant asymmetry.

### 2.6. Statistical Analysis

Pooled hazard ratios (HR) meta-analyses were used to compare the two different treatment arms on the basis of all primary outcomes. The 95% confidence intervals of HR reported in the individuals studies were used to calculate the standard errors applied in the meta-analyses. The calculations were made using the suggestions available in the Cochrane Handbook for Systematic Reviews of Interventions [17].

The choice between a random- and fixed-effects model was made based on statistical heterogeneity (Cochran’s Q and I^2^) indices as follows: if I^2^ > 50% and/or PQ < 0.10 the random effects model was implemented, otherwise the fixed effects model was used [18]. Statistical significance was set at *p* < 0.05. The funnel plots were visually inspected for the presence of publication bias. Sensitivity analyses, including leave-one-out procedures by study and by CDK4/6 inhibitor, were conducted to evaluate the robustness of the pooled results. All analyses were performed using Review Manager (Rev-Man) [Computer program], Version 5.3. Copenhagen: The Nordic Cochrane Centre, The Cochrane Collaboration, 2014.

## 3. Results

### 3.1. Database Search

**Figure 1:**
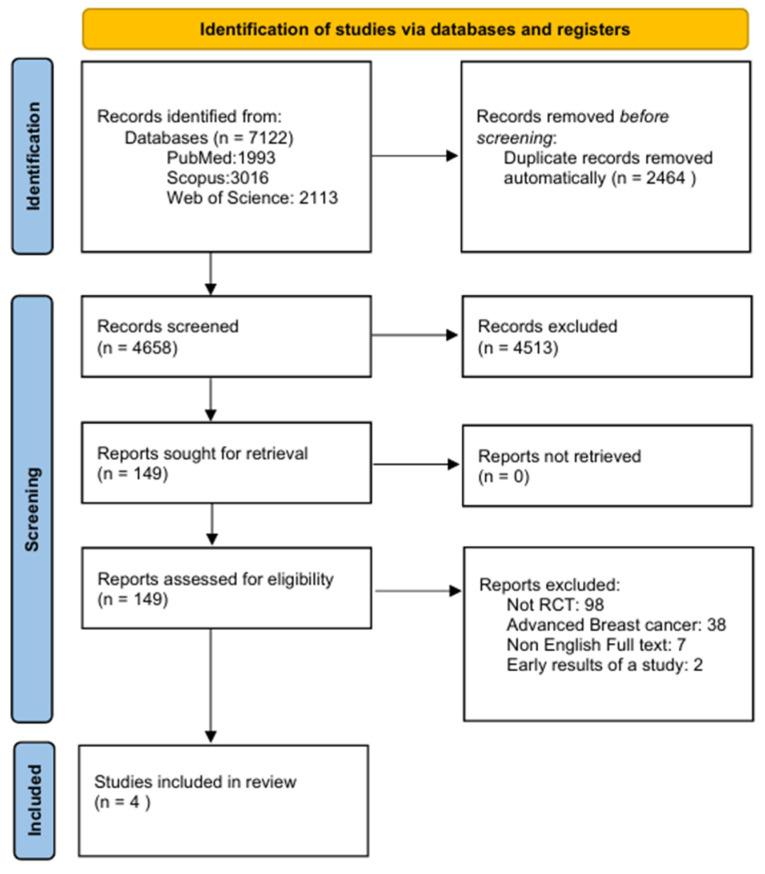
PRISMA flowchart presenting the study selection process.

Following an extensive search of the databases, 7122 records were identified. Of these, 2464 records were automatically removed as duplicates, resulting in 4658 records eligible for screening. Subsequently, 4513 records were excluded as they were considered irrelevant to the scope of the meta-analysis or did not fulfill the predefined inclusion criteria. A total of 149 reports satisfied the initial inclusion criteria and were successfully retrieved for additional assessment. Following detailed evaluation: 98 reports were excluded as they were not RCTs, 38 focused on advanced breast cancer, 7 were excluded due to the lack of an English full text and 2 were excluded as they reported early results of a study (File S1). Ultimately, based on eligibility criteria, four studies were identified and included in the systematic review and meta-analysis (Figure 1).

### 3.2. Study Characteristics

#### 3.2.1. Demographics

The studies included a total of 17,749 patients, of whom 8872 received treatment with a CDK4/6 inhibitor and endocrine therapy, while 8877 received only endocrine therapy and comprised the control group (Table 1). The median age of patients in the intervention group of the included studies ranged from 49 to 52 years, while in the control group it ranged from 48 to 52 years. The percentage of male participants in the intervention group of the included studies was between 0.0% and 0.8%, compared to 0.0% to 0.7% in the control group [10,11,12,13].

#### 3.2.2. Clinical Information

The duration of CDK4/6i administration was 12 months in the PENELOPE-B trial, 24 months in the monarchE and PALLAS trials, and 36 months in the NATALEE trial. In all four studies, patients received endocrine therapy for at least 5 years. Follow-up results were reported at 31 months for the PALLAS trial, 42.8 months for the PENELOPE-B trial, 44.2 months for the NATALEE trial, and 54 months for the monarchE trial.

Regarding menopausal status in the intervention group of the included studies, the proportion of premenopausal women ranged from 43% to 47.5%, compared to 44% to 51.1% in the control group. The proportion of postmenopausal women in the intervention group of the included studies ranged from 52.5% to 57%, while in the control group it ranged from 48.9% to 56% [10,11,12,13].

Concerning nodal status at the time of diagnosis, the percentage of patients with N0 status in the intervention group was 27.2% in the NATALEE trial, 10.5% in the PENELOPE-B trial, and 12.7% in the PALLAS trial. The corresponding percentages in the control group were 28.9% for NATALEE, 11.5% for PENELOPE-B, and 13.4% for PALLAS. The percentage of patients with N1 status in the intervention group was 40% in the monarchE trial, 41.2% in the NATALEE trial, 68.6% in the PENELOPE-B trial, and 49.6% in the PALLAS trial. The corresponding percentage in the control group was 40% for monarchE, 41.1% for NATALEE, 67.4% for PENELOPE-B, and 49% for PALLAS. The percentage of patients in the intervention group with N2-3 status was 59% for monarchE, 18.9% for NATALEE, 20.9% for PENELOPE-B, and 37.7% for PALLAS. The corresponding percentage in the control group was 60% for monarchE, 18.3% for NATALEE, 21.4% for PENELOPE-B, and 37.5% for PALLAS [10,11,12,13].

Regarding Ki-67, in the monarchE study 34% of patients in both the intervention and control groups had a Ki-67 level of <20%, while 45% in the intervention group and 44% in the control group had a Ki-67 of ≥20%. In the NATALEE study, 56% of patients in both the intervention and control groups had a Ki-67 level of ≤20%, while 43% had a Ki-67 level of >20%. In the PENELOPE-B study, 74% of patients in both groups had a Ki-67 level of ≤15%, while 25% had a Ki-67 level of >15% (Table 2).

Overall, the included trials differed notably in their patient populations and design characteristics. Specifically, monarchE and PENELOPE-B enrolled exclusively patients with high-risk features, while PALLAS and NATALEE included a broader early breast cancer population with lower-risk disease. The duration of CDK4/6 inhibitor administration also varied considerably, ranging from 12 months in PENELOPE-B to 36 months in NATALEE, reflecting differences in treatment strategies and study objectives.

#### 3.2.3. Adverse Effects

A qualitative synthesis of adverse events from the included trials is presented below. In the PALLAS study, no new adverse events were reported with the use of palbociclib. However, 99.5% of patients receiving palbociclib plus ET experienced at least one adverse event, with 13.0% reporting serious adverse events. In comparison, 89.7% of patients in the ET-only group experienced adverse events, and 7.9% reported serious adverse events. The most common adverse events in the palbociclib plus ET group were neutropenia (83.5%), leukopenia (55.1%), and fatigue (41.0%), all of which were less frequent in the ET-only group (5.0%, 7.5%, and 19.3%, respectively) [12].

In the PENELOPE-B study, almost all patients, except one in each treatment arm experienced at least one adverse event. Grade 3–4 adverse events were significantly more frequent in the palbociclib plus ET group (79.6%) compared to the ET-only group (20.1%). The most common adverse events in the palbociclib plus ET group, occurring with higher frequency than in the control group, included neutropenia of any grade (95.7% vs. 23.4%), with grade 3–4 neutropenia reported in 70.0% of patients compared to 1.0% in the control group; leukopenia of any grade (99.2% vs. 69.9%), with grade 3–4 leukopenia occurring in 56.1% vs. 0.7%; thrombocytopenia of any grade (56.6% vs. 16.2%); anemia (73.9% vs. 30.3%); hypocalcemia (35.2% vs. 24.4%); fatigue (66.4% vs. 51.1%); stomatitis (27.5% vs. 8.7%); constipation (22.1% vs. 13.7%); cough (9% vs. 16.2%); and infection (59.9% vs. 51.1%) [11].

In the NATALEE study, the most frequently reported adverse events in the ribociclib plus ET group were neutropenia of any grade (62.8% vs. 4.5%), with grade ≥ 3 neutropenia reported in 44.4% of patients compared to 0.9% in the control group; arthralgia (38.8% vs. 44.4%); liver-related adverse events (26.7% vs. 11.4%); nausea (23.5% vs. 7.9%); headache (22.9% vs. 17.2%); and fatigue (22.8% vs. 13.5%). A noteworthy proportion of patients in the intervention group also experienced diarrhea (14.6% vs. 0.5%) [10].

Finally, in the monarchE study, at least one adverse event was reported by 97.5% of patients receiving abemaciclib plus ET, compared to 82.8% in the ET-only group. The most common adverse event in the intervention group was diarrhea (82.2% vs. 7.1%), followed by neutropenia (44.6% vs. 5.0%), leukopenia (36.8% vs. 6.1%), fatigue (38.5% vs. 15.5%), and abdominal pain (33.9% vs. 8.1%) [13].

### 3.3. Outcomes

#### 3.3.1. iDFS

**Figure 2:**
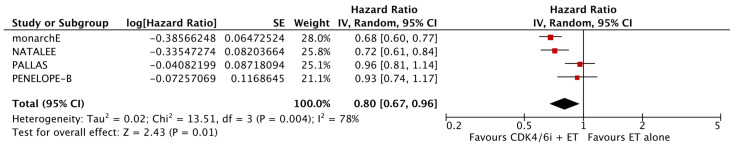
Forest plot showing the effect of CDK4/6 inhibitors on iDFS.

**Figure 3:**
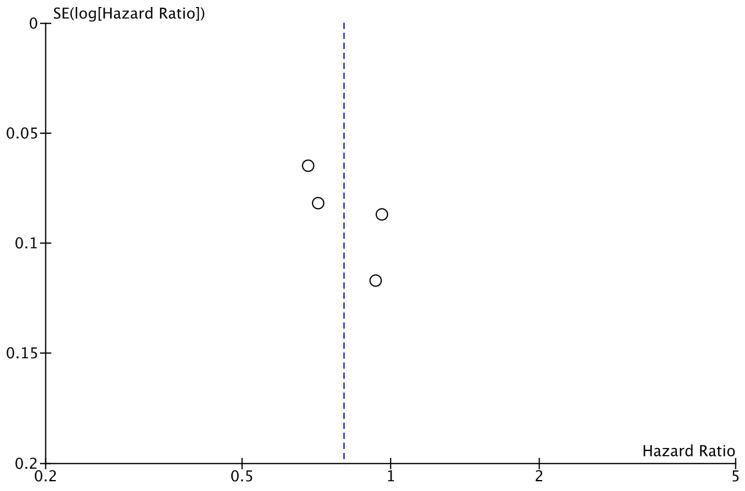
Funnel plot showing the distribution of studies included in the iDFS analysis.

As shown in Figure 2, the analysis demonstrated that the addition of CDK4/6 inhibitors led to a significant reduction in iDFS events in the intervention group compared to the group receiving endocrine therapy alone (HR: 0.80; 95% CI: 0.67–0.96; *p*: 0.01; I^2^: 78%). Visual assessment of the funnel plots did not reveal evidence for significant publication bias (Figure 3).

#### 3.3.2. DRFS

**Figure 4:**
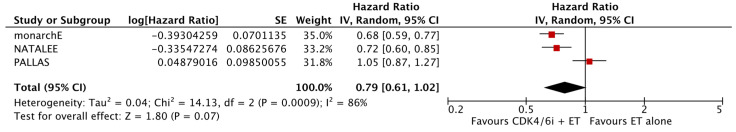
Forest plot showing the effect of CDK4/6 inhibitors on DRFS.

**Figure 5:**
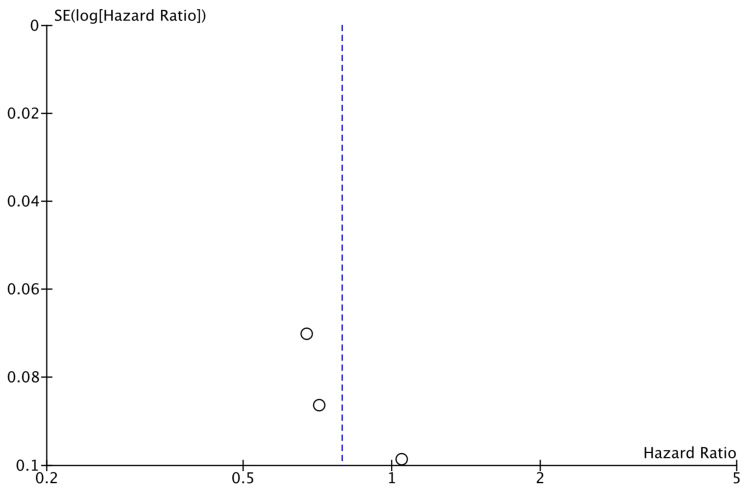
Funnel plot showing the distribution of studies included in the DRFS analysis.

The analysis of data from the three studies for which DRFS data were available did not reveal a significant difference between the two study groups (HR: 0.79; 95% CI: 0.61–1.02; *p*: 0.07; I^2^: 86%) (Figure 4). No reveal evidence for significant publication bias was noted (Figure 5).

#### 3.3.3. OS

**Figure 6:**
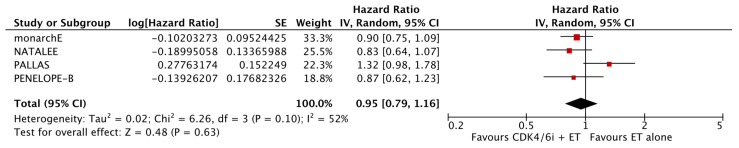
Forest plot showing the effect of CDK4/6 inhibitors on OS.

**Figure 7:**
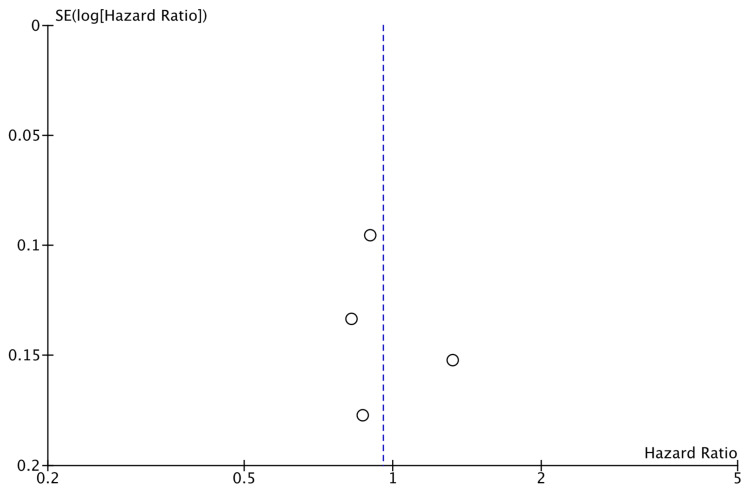
Funnel plot showing the distribution of studies included in the OS analysis.

The analysis of data from the four studies for which OS data were available did not reveal a significant improvement with the addition of CDK4/6i (HR: 0.95; 95% CI: 0.79–1.16; *p*: 0.63; I^2^: 52%) (Figure 6). Again, there was no evidence for significant publication bias (Figure 7).

### 3.4. Sensitivity Analyses

Sensitivity analyses and leave-one-out analyses did not significantly affect our results (Table 3 and Table 4).

A leave-one-out sensitivity analysis was conducted based on the specific CDK4/6 inhibitor used. For iDFS, exclusion of ribociclib (HR 0.86, 95% CI 0.66–1.07; I^2^ = 84%; *p* = 0.16) or abemaciclib (HR 0.86, 95% CI 0.70–1.04; *p* = 0.12) did not materially alter the results. This analysis was not feasible for DRFS due to data availability from only three trials. For OS, exclusion of ribociclib (HR 1.01, 95% CI 0.78–1.29; I^2^ = 60%; *p* = 0.97) or abemaciclib (HR 0.98, 95% CI 0.73–1.32; I^2^ = 66%; *p* = 0.91) yielded consistent estimates, confirming the robustness of the pooled outcomes.

### 3.5. Risk of Bias

**Figure 8:**
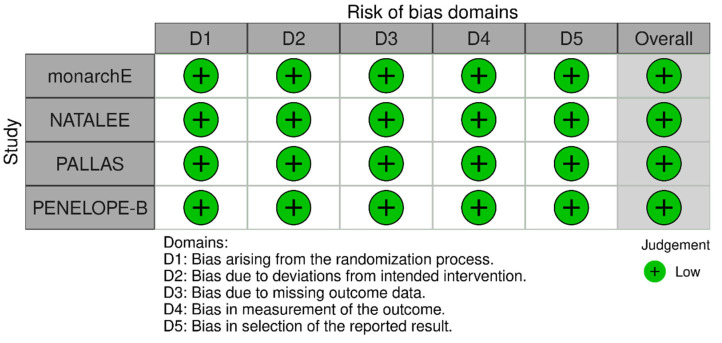
Risk of bias assessment for the included studies.

The Risk of Bias assessment revealed low risk of bias for all included studies (Figure 8).

## 4. Discussion

This meta-analysis incorporated data from four pivotal clinical trials (Natalee, monarchE, PENELOPE-B, and PALLAS) that assessed the efficacy of CDK4/6 inhibitors in the adjuvant treatment of HR+/HER2− early breast cancer. The key outcomes of this meta-analysis indicate that CDK4/6 inhibitors significantly improve iDFS, while a clear trend toward benefit is also observed in DRFS, suggesting a potentially clinically meaningful effect that has not yet reached statistical significance. This borderline result (HR 0.79; 95% CI: 0.61–1.02) likely reflects data immaturity, trial heterogeneity, and limited follow-up rather than a true absence of effect. In contrast, no significant difference in OS has been demonstrated to date.

Although all of the aforementioned trials evaluate the efficacy of CDK4/6 inhibitors in the adjuvant setting, their findings differ regarding the extent of benefit observed, emphasizing the importance of examining factors that may explain these variations. These differences may be attributed to variations in trial design, patient populations, drug properties, and treatment protocols. For example, the monarchE trial specifically enrolled high-risk patients, with 74.1% having stage III disease and 38.7% presenting with grade 3 tumors, while the PALLAS trial included a more diverse population, of whom only 48.9% and 29.3% in these respective categories [19,20]. However, subgroup analyses in the PALLAS trial showed no significant differences between high-risk and lower-risk patients, indicating that high-risk status alone may not account for the variations in outcomes observed [19]. Additionally, the monarchE trial included more patients with high Ki-67 expression and lacked standardized imaging to rule out subclinical metastatic disease, which may have led to the inclusion of patients already responding to systemic therapy. This could be another possible explanation for the better results observed in the monarchE trial [20].

There were also important differences in how the treatment was administered. In monarchE, abemaciclib was used continuously, while palbociclib in PALLAS and ribociclib in NATALEE followed a 3 weeks on, 1 week off schedule. Continuous treatment may be more effective in stopping dormant cancer cells from reactivating, which is thought to play a role in early breast cancer [20,21]. Additionally, abemaciclib demonstrates unique pharmacological features, such as a wider range of kinase inhibition and stronger activity against CDK4 than CDK6. Studies have shown that breast cells have a higher overall dependency on CDK4 compared to CDK6 [22]. These features may help explain why abemaciclib performs differently in the adjuvant setting compared to palbociclib and ribociclib [23,24].

In PALLAS, a high rate of early treatment discontinuation (30% of patients stopped within the first year) was linked to strict protocol requirements. However, post hoc analyses found no correlation between palbociclib exposure or duration and improved iDFS, ruling out inadequate drug exposure as a cause for its negative results [19,21]. It is worth noting that a similar discontinuation rate was observed in the NATALEE trial (36%), whereas in monarchE, which reported more favorable outcomes, the corresponding rate was only 17%. Moreover, neither the monarchE nor the PALLAS trial showed a benefit in overall survival, and early results from monarchE even indicated a hazard ratio temporarily favoring the control group. These findings highlight concerns about potential overtreatment, since many patients in these trials might never experience a relapse, and emphasize the importance of developing biomarkers to better identify those who are most likely to benefit. Exposing such patients to CDK4/6 inhibitors increases toxicity, treatment costs, and may reduce their ability to benefit from the same therapies upon relapse [20].

Although CDK4/6 inhibitors have demonstrated clinical benefits in certain patient groups, their application in the adjuvant setting raises important considerations regarding potential toxicities, economic burden, and consequences for post-relapse therapy. Real-world data suggest that hematologic and gastrointestinal adverse events frequently lead to dose adjustments or treatment discontinuation, with neutropenia and diarrhea reported as the most commonly reported events [25]. Treatment discontinuation rates varied among trials, ranging from 17% in monarchE, 18–20% in PENELOPE-B, and reaching 30–36% in PALLAS and NATALEE, reflecting differences in tolerability and adherence. In addition to toxicity, long term use of CDK4/6 inhibitors contributes to a substantial financial burden, including drug acquisition, monitoring, and supportive care costs [26,27]. Moreover, accumulating data indicate that previous adjuvant use of CDK4/6 inhibitors may influence treatment response and resistance mechanisms after recurrence, underscoring the importance of strategic sequencing and biomarker-guided patient selection [28,29]. Overall, these considerations underscore the importance of weighing the potential clinical benefits of CDK4/6 inhibitors against considerations of toxicity, cost-effectiveness, and their impact on subsequent treatment approaches.

The differences in trial results also raise questions about the potential for false positive or negative findings. While monarchE’s favorable results may represent a false positive due to potential biases in patient selection (for instance, the lack of standardized imaging and the inclusion of patients with high Ki-67 expression, as mentioned above), PALLAS and PENELOPE-B may reflect true negatives, challenging the role of CDK4/6 inhibitors in the adjuvant setting [19,20]. Furthermore, the emergence of resistant clones from early drug exposure, combined with the absence of reliable biomarkers for identifying likely responders, underscores the need for more refined trial methodologies. Current genomic assays remain inadequate for stratification in both the metastatic and adjuvant settings [20,21].

In summary, the data reflect how difficult it is to define the exact role of CDK4/6 inhibitors in early-stage breast cancer treatment. More research is needed to explain the variable results seen across trials and to find reliable ways to choose the patients who are most likely to benefit.

The main strength of this meta-analysis is that it includes the latest data from two clinical trials (NATALEE, monarchE). Additionally, a risk of bias assessment was conducted to evaluate whether there were any variations in the results. Regarding the limitations of the meta-analysis they mostly derive from the design of the included studies. It is important to note that there is considerable heterogeneity among the studies. The medications administered to the patients, the timing and dosages of administration, the follow-up duration, lymph node status, and the Ki-67 percentage differ across the studies. Due to the heterogeneity of the available data, it was not feasible to conduct formal subgroup analyses by CDK4/6 inhibitor type, treatment duration, or patient risk category, including a focused analysis on high-risk populations. This limitation restricts the ability to determine whether the observed benefit is driven by specific agents or patient subsets. Moreover, the relatively short follow-up duration in some trials limits the conclusions that can be drawn regarding long-term survival outcomes.

We included updated data from conference abstracts, which could be considered a limitation; however, these abstracts report results from trials that have already been fully published in high-impact journals, and we used them solely to incorporate the most recent follow-up data.

## 5. Conclusions

In conclusion, this meta-analysis examined the role of CDK4/6 inhibitors combined with endocrine therapy in the adjuvant treatment of HR+/HER2− early breast cancer. Our results demonstrate that CDK4/6 inhibitors provide a clear benefit in iDFS primarily by delaying local or regional recurrences. Its effect on DRFS and OS remains limited. These findings emphasize the value of selecting high-risk patients who are most likely to benefit from CDK4/6i in the adjuvant setting. Overall, the results support the role of CDK4/6i as a valuable addition to adjuvant therapy, while emphasizing the need for longer follow-up and further studies to better define their long-term impact and optimal use in clinical practice.

## Figures and Tables

**Table 1:** Table presenting the main characteristics of the included studies.

Study ID YOP	monarchE 2024	NATALEE 2024	PENELOPE-B 2021	PALLAS 2021
**Number** **(intervention)**	2808	2549	631	2884
**Number** **(control)**	2829	2552	619	2877
**Male** **(intervention)**	21	11	0	17
**Male** **(control)**	15	9	0	19
**Female** **(intervention)**	2787	2538	631	2867
**Female** **(control)**	2814	2543	619	2858
**Age** **(intervention)** **Median (IQR)**	51 (44–60)	52 (24-90)	49 (22–76)	52 (45–61)
**Age** **(control)** **Median (IQR)**	51 (44–60)	52 (24-89)	48 (19–79)	52 (45–60)
**Drugs** **(intervention)**	Abemaciclib + ET	Ribociclib + ET	Palbociclib + ET	Palbociclib + ET
**Drugs** **(control)**	ET	ET	ET	ET
**Duration** **(intervention)**	2 years	3 years	1 year	2 years
**Duration** **(control)**	5 years	5 years	5 years	5 years
**Dosage** **(intervention)**	150 mg	400 mg	125 mg	125 mg
	twice daily	once daily	once daily	once daily

All data presented in this table were extracted from the primary publications of the respective trials: NATALEE [10], PENELOPE-B [11], PALLAS [12] and monarchE [13].

**Table 2:** Table presenting the patient characteristics of the included studies.

Study ID YOP	monarchE 2024	NATALEE 2024	PENELOPE-B 2021	PALLAS 2021
**Pre-menopausal** **(intervention)**	1221	1115	300	1303
**Pre-menopausal** **(control)**	1232	1123	316	1323
**Post-menopausal** **(intervention)**	1587	1424	331	1562
**Post-menopausal (control)**	1597	1420	303	1534
**Grade (intervention)**	Grade 1: 209 Grade 2: 1377 Grade 3: 1086	Grade X: 30 Grade 1: 218 Grade 2: 1458 Grade 3: 521 Not assessed: 292 Data missing: 30	Grade 1: 31 Grade 2: 355 Grade 3: 237 Missing: 8	Grade X: 118 Grade 1–2: 1926 Grade 3: 836 Unknown: 4
**Grade (control)**	Grade 1: 216 Grade 2: 1395 Grade 3: 1064	Grade X: 32 Grade 1: 240 Grade 2: 1451 Grade 3: 549 Not assessed: 258 Data missing: 22	Grade 1: 36 Grade 2: 330 Grade 3: 245 Missing: 8	Grade X: 137 Grade 1–2: 1971 Grade 3: 769 Unknown: 0
**Lymph node status (intervention)**	Ν1: 1118 Ν2: 1107 Ν3: 575	NX: 272 N0: 694 N1: 1050 N2 or N3: 483 Data missing: 50	N0: 66 N1: 433 N2: 80 N3: 52	Ν0: 365 Ν1: 1431 Ν2: 700 Ν3: 386 ΝΧ: 1 Unknown: 1
**Lymph node status (control)**	Ν1: 1142 Ν2: 1126 Ν3: 554	NX: 264 N0: 737 N1: 1049 N2 or N3: 467 Data missing: 35	N0: 71 N1: 417 N2: 82 N3: 49	ΝΧ: 0 Ν0: 385 Ν1:1411 Ν2:709 Ν3: 372 Unknown: 0
**Ki 67 (intervention)**	<20%: 953 ≥20%: 1262	≤20%: 1199 >20%: 920	≤15%: 470 >15%: 161	Not available
**Ki 67 (control)**	<20%: 974 ≥20%:1233	≤20%: 1236 >20%: 937	≤15%: 461 >15%: 158	Not available
**Follow up time** **(intervention)** **Median (IQR)**	Median (IQR): 54 (49–59)	Median: 44.2 months	Median: 42.8 months	Median (IQR): 31 (24.5–37.3) months
**Follow up time** **(control)**	54 (49–59)	44.2 months	42.8 months	31 (24.5–37.3) months

All data presented in this table were extracted from the primary publications of the respective trials: NATALEE [10], PENELOPE-B [11], PALLAS [12] and monarchE [13].

**Table 3:** Sensitivity analyses of the included studies.

Outcome	Preferred Model Used Outcome	Alternative (Sensitivity) Model
**iDFS**	RE HR 0.80; 95% CI: 0.67–0.96; *p* = 0.01; I^2^ = 78%	FE HR 0.77; 95% CI: 0.71–0.84; *p* < 0.001; I^2^ = 78%
**DRFS**	RE HR 0.79; 95% CI: 0.61–1.02; *p* = 0.07; I^2^ = 86%	FE HR 0.76; 95% CI: 0.69–0.84; *p* < 0.001; I^2^ = 86%
**OS**	RE HR 0.95; 95% CI: 0.79–1.16; *p* = 0.63; I^2^ = 52%	FE HR 0.94; 95% CI: 0.83–1.07; *p* = 0.36; I^2^ = 52%

**Table 4:** Leave-one-out analyses of the included studies.

Outcome	Leaving monarchE Out	Leaving NATALEE Out	Leaving PALLAS Out	Leaving PENELOPE-B Out
	Model used Outcome	Model used Outcome	Model used Outcome	Model used Outcome
iDFS	RE HR 0.86; 95% CI: 0.70–1.04; *p* = 0.12; I^2^ = 71%	RE HR 0.84; 95% CI: 0.66–1.07; *p* = 0.16; I^2^ = 84%	RE HR 0.75; 95% CI: 0.6–0.88; *p* < 0.001; I^2^ = 64%	RE HR 0.77; 95% CI: 0.63–0.94; *p* = 0.01; I^2^ = 81%
OS	RE HR 0.98; 95% CI: 0.73–1.32; *p* = 0.91; I^2^ = 66%	RE HR 1.01; 95% CI: 0.78–1.29; *p* = 0.97; I^2^ = 60%	FE HR 0.88; 95% CI: 0.76–1.01; *p* = 0.06; I^2^ = 0%	RE HR 0.98; 95% CI: 0.77–1.25; *p* = 0.87; I^2^ = 67%

## Data Availability

No new data were created or analyzed in this study. Data sharing is not applicable to this article.

## Supplementary Materials

The following supporting information can be downloaded at https://www.mdpi.com/article/10.3390/cancers17213538/s1; Table S1: PRISMA_checklist. File S1: Summary of reasons for exclusion.

## Abbreviations

The following abbreviations are used in this manuscript:

CDK4/6i	CDK4/6 inhibitors
ET	Endocrine therapy
DRFS	Distant recurrence-free survival
iDFS	Invasive disease-free survival
OS	Overall survival
HR+	Hormone Receptor-positive
HER2−	Human Epidermal Growth Factor 2-negative
RCTs	Randomized controlled trials
CDKs	Cyclin-dependent kinases
ER+	Estrogen receptor positive
Rb	Retinoblastoma protein
a/mBC	Advanced or metastatic breast cancer
PFS	Progression-free survival
ABC	Advanced breast cancer
DFS	Disease-free survival
HR	Hazard ratios
