# Supplementary material for: CDK4/6 Inhibitors Plus Endocrine Therapy in Early-Stage HR+/HER2− Breast Cancer: Updated Meta-Analysis of Phase III Trials
Source: Cancers (Basel). 2025 Nov 1;17(21):3538. doi: 10.3390/cancers17213538 (PMC12608974; doi:10.3390/cancers17213538)
Supplement: Supplementary file 1 [file cancers-17-03538-s001.zip › File S1.pdf]

File S1: Summary of Reasons for Exclusion

Reason for Exclusion	Number of trials
Non RCT	98
Advanced Breast cancer	38
Non English Full Text	7
Early results of a study	2
